# Retinal Degeneration Progression Changes Lentiviral Vector Cell Targeting in the Retina

**DOI:** 10.1371/journal.pone.0023782

**Published:** 2011-08-25

**Authors:** Maritza Calame, Maité Cachafeiro, Stéphanie Philippe, Karine Schouwey, Meriem Tekaya, Dana Wanner, Chamsy Sarkis, Corinne Kostic, Yvan Arsenijevic

**Affiliations:** 1 Unit of Gene Therapy and Stem Cell Biology, Service of Ophthalmology, Jules-Gonin Eye Hospital, University of Lausanne, Lausanne, Switzerland; 2 NewVectys SAS, Paris, France; 3 Team of Biotherapy and Biotechnology, CRICM, UPMC-Paris6 UMR_S 975, INSERM U975, CNRS UMR 7225, Paris, France; Institut de la Vision, France

## Abstract

In normal mice, the lentiviral vector (LV) is very efficient to target the RPE cells, but transduces retinal neurons well only during development. In the present study, the tropism of LV has been investigated in the degenerating retina of mice, knowing that the retina structure changes during degeneration. We postulated that the viral transduction would be increased by the alteration of the outer limiting membrane (OLM). Two different LV pseudotypes were tested using the VSVG and the Mokola envelopes, as well as two animal models of retinal degeneration: light-damaged Balb-C and Rhodopsin knockout (*Rho*-/-) mice. After light damage, the OLM is altered and no significant increase of the number of transduced photoreceptors can be obtained with a LV-VSVG-Rhop-GFP vector. In the *Rho*-/- mice, an alteration of the OLM was also observed, but the possibility of transducing photoreceptors was decreased, probably by ongoing gliosis. The use of a ubiquitous promoter allows better photoreceptor transduction, suggesting that photoreceptor-specific promoter activity changes during late stages of photoreceptor degeneration. However, the number of targeted photoreceptors remains low. In contrast, LV pseudotyped with the Mokola envelope allows a wide dispersion of the vector into the retina (corresponding to the injection bleb) with preferential targeting of Müller cells, a situation which does not occur in the wild-type retina. Mokola-pseudotyped lentiviral vectors may serve to engineer these glial cells to deliver secreted therapeutic factors to a diseased area of the retina.

## Introduction

Research in gene therapy for eye diseases has become increasingly interesting with the development of vectors efficient in targeting different cell types of the retina or the cornea. Among different vector families, the AAV vectors and the lentiviral vectors are widely used for either a gene replacement or a neuroprotective strategy. Depending on the serotype, AAV vectors can preferentially target the retinal pigmented epithelium (RPE[1], [2]), or photoreceptors[3], [4], whereas lentiviral vectors predominantly transduce the RPE cells[5], [6] and Müller cells at the injection site[7]. AAV-based gene therapy has shown many successes in gene replacement treatments for animal models of autosomal inherited retinal degeneration[8], whereas the HIV1-derived lentiviral vectors (LVs) were efficient against diseases affecting the RPE[1], [9]–[11], in retinal ganglion cells when injured[12], and in photoreceptors when the cells are targeted early during development[13]. Indeed, the HIV1-derived lentiviral vector can target photoreceptors just after birth and some days later when no physical barrier is formed yet between the RPE and the photoreceptors, and, between photoreceptors themselves[14]. The equine lentiviral vector seems to have the same behaviour, with a slightly increased capacity to transduce photoreceptors[15], [16], still improved using the Rabbies-G envelope[15].

Most of the studies on vector tropism were undertaken in normal retinas or at an early stage of retinal degeneration and little is known concerning the ability of the vectors to transduce retinal cells at advanced stages of retinal degeneration. During disease progression, the retina undergoes a major structural remodelling, thus creating a different environment that may impact vector diffusion and transduction capabilities. Indeed, the retina shows important changes in neurons[17], [18] and glia such as gliosis, rupture of the outer limiting membrane[19], [20] (OLM), cell loss, and remodelling of synaptic connexions (for review[21]). These retinal modifications were mainly studied with the goal of improving cell integration after transplantation into the retina. Cell transplantation studies revealed that the OLM and extracellular matrix proteins impair the passage of cells from the subretinal space towards the inner retina [22], [23]. For instance, the loss of the Crumb protein which participates to the cohesion of the OLM, facilitates the migration of transplanted primary retinal cells into the outer nuclear layer[22]. Concerning gene transfer, a recent interesting work shows that, after intravitreal injection, a Cy3-labelled-AAV vector diffuses into the retina of a rat model of retinal degeneration, whereas no diffusion into this tissue was observed in wild-type animals. Moreover, cells of all layers, including photoreceptors can be targeted, the efficiency remaining to be improved. This study shows the possibility to target retinal cells, when the structural change of the inner-limiting membrane occurs during retinal disease, [24].

Hypothesizing that the structural remodelling taking place during the course of retinal degeneration could limit the barrier mechanism, we have investigated in the present study whether HIV1-derived lentiviral vectors have a capacity to diffuse through retinal layers and transduce various cell types when injected into degenerating retinas. We focused our study on two different animal models of photoreceptor loss, one acutely induced, the light damage model, the other modelling an inherited disease, the *Rhodopsin* knockout mouse (*Rho*-/-). These experiments were performed using HIV1-derived vectors pseudotyped with the pantropic vesicular stomatitis virus glycoprotein (VSV-G) or the Lyssavirus Mokola glycoprotein (Mok-G) previously shown to preferentially target glial cells in the central nervous system[25].

## Results

**Figure 1:**
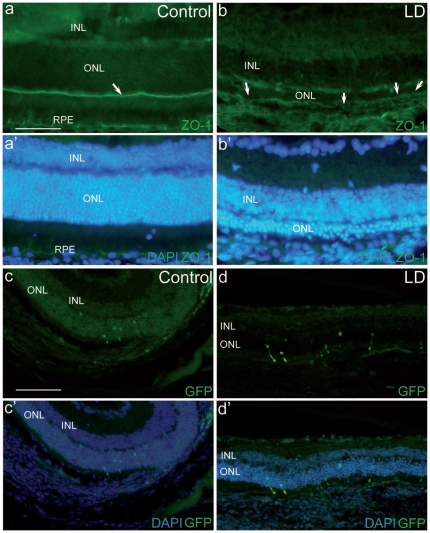
The OLM is altered after light damage (LD). (a) ZO-1 is homogeneously expressed along the OLM in untreated mouse (a, arrow), whereas 36 hours after light damage, disruption of the protein labelling is apparent (b, arrows). Note also the reduced expression of ZO-1 after light damage. The slices were processed simultaneously for the immuhistochemistry labelling and pictures were taken with the same exposure time (700 ms). (a′, b′) show the nuclear staining of the respective retinas. Note that the ONL is markedly reduced in the light-damaged retina (b′). Representative pictures of wild-type mouse retina (c) and light damaged retina (d) subretinally injected with LV-VSVG-Rhop-GFP. Few photoreceptors (green) were transduced in both cases. No significant differences were observed between groups (see text). The transduced area was confined to the injection site. (c′ and d′) Merged picture of GFP expressing cells with DAPI staining. Calibration bar in (a): 50 µm (for a, a′,b and b′). Calibration bar in (c): 100 µm (for c, c′, d and d′).

To investigate whether the lentiviral vector (LV) targets the neuroretina better during retinal degeneration, we first examined the integrity of the OLM in an acute model of retinal degeneration, the light damage model. Mice were submitted to 5000 lux during one hour and the retina fixed 36 hours later, at the apoptosis peak, for immunohistochemistry investigation. Zona-occludin-1 (ZO-1) is a major component stabilizing the structure of adherent junctions and was shown to be altered in certain neurodegenerative processes including the *Rhodopsin* knockout (*Rho*-/-) mouse[19], [20]. ZO-1 participates to tight junctions as well as adherent junctions and, with the help of other members of the cadherin, catenin and occludin family members, forms a hermetic barrier at the OLM level. These proteins are deregulated in the *Rho*-/- retina [19], [20]. In control mice, a linear continuous labelling of ZO-1 can be observed at the level of the OLM (Figure 1a). In mice subjected to light damage, several disruptions of the ZO-1 linear staining are evident in the central part of the retina with a reduced expression (Figure 1b), but not in the periphery where no toxic effects occur.

In consequence, in order to assess the influence of these structural remodellings of the retina on lentiviral transduction and diffusion, we injected groups of mice 36 hours after light damage with a LV driving the expression of GFP under the *Rhodopsin* promoter and pseudotyped with a VSVG envelope (LV-VSVG-Rhop-GFP) to first investigate whether the photoreceptors can be targeted in this model. The number of GFP-positive photoreceptors per slice was counted 7 days later. No significant differences were observed between groups (Figure 1 c and d, controls: 71±37.5 and light damage: 71±22.5 GFP cells/200 µm, n = 4 and 6 respectively). The rapid loss of outer segments and photoreceptors after the light damage induction may be in part responsible for the impaired efficacy of LV penetration into the ONL in this model. A constant loss of photoreceptors during the first days after light damage may have eliminated the cells that were preferentially transduced after the OLM rupture. We also investigated whether the OLM was reinforced by a Müller cell process (explaining the poor vector diffusion), but no glial cell reaction at the ONL level was observed 36 hours after light damage (data not shown).

**Figure 2:**
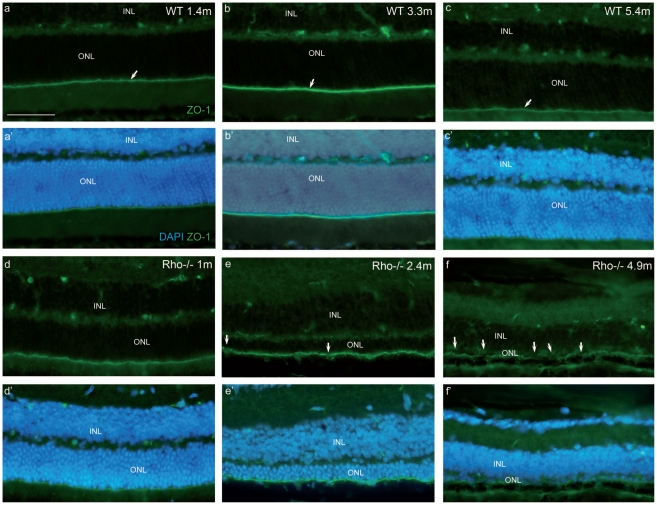
ZO-1 expression in the OLM is altered in old *Rho*-/- mice. ZO-1 immunolabelling of wild-type (WT) retina is homogenous throughout the retina in 1.4, 3.3 and 5.4 month-old mice respectively (a, b, c, arrows), but the labelling changes with age. In *Rho*-/- mice, ZO-1 distribution is normal at 1 month of age (d), starts to show few alterations at 2.4 months (e, arrows) and is often disrupted at 4.9 months (f, arrows). (a′, b′, c′, d′, e′, f′) represent the nuclei of the above pictures. Note the progressive photoreceptor loss in the *Rho*-/- mice (d′,e′,f′). INL: inner nuclear layer, ONL: outer nuclear layer. All retina slices of this figure were processed simultaneously and pictures were taken with the same exposure time (400 ms). Calibration bar in (a): 50 µm (for all pictures).

**Figure 3:**
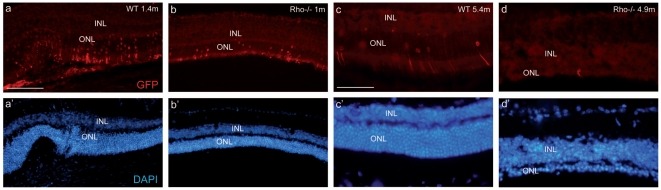
Low photoreceptor transduction with the LV-VSVG-Rhop-GFP in the *Rho*-/- retina. Injections of the vector into 1.4 and 5.4 month old wild-type (WT) retinas transduce few photoreceptors (red, a, c). Similar results were observed in the 1 month-old *Rho*-/- mice (b), and the transduction efficiency is even worse at 4.9 month (d). (a′, b′, c′, d′) represent the nuclei (DAPI staining) of the above pictures. INL: inner nuclear layer, ONL: outer nuclear layer. Calibration bar in (a) 100 µm (for a, a′, b and b′); calibration bar in (c): 50 µm (for c, c′, d and d′).

We then decided to test the same vector in an animal model of slow retinal degeneration process to avoid the problem of rapid photoreceptor loss. The vector diffusion and transduction capacity was investigated in young adults as well as more mature *Rhodopsin* knockout mice (*Rho*-/-), which have no phototransduction in their rods and show a rod loss starting at around one month. The integrity of the OLM was first investigated, previous results having already reported alteration of the ZO-1 localization in the *Rho*-/- mouse[19], [20]. At 1 and 1.4 months of age, the retinas of the *Rho*-/- and wild type controls show no differences respectively, whereas few alterations of the ZO-1 structure can be detected at 2.4 months (Figure 2e). At 4.9 months in *Rho*-/- retinas, ZO-1 labelling presents a multitude of discontinuous regions revealing a profound change of the OLM structure (Figure 2f). These results confirm previous observations[19] also showing that the integrity of the OLM is altered in this model, although in our hands the *Rho*-/- retinas show a delayed alteration of ZO-1 distribution in the OLM. Nonetheless, our results confirm that the OLM barrier is partially modified, potentially allowing a better diffusion of lentiviral vectors when injected subretinally. To test this hypothesis and to reveal transduced photoreceptors, the LV-VSVG-Rhop-GFP was injected into young adults (1 month old), before the disruption of the ZO-1 expression, and advanced-stage (4.9 months old) *Rho*-/- mice, when the OLM structure is altered, as well as in age-matched wild type C57/Bl6 control mice (WT). In young adult animals (Figure 3a and b), we observed retinal transduction near the site of injection in *Rho*-/- or C57/Bl6 mice. Few photoreceptors were transduced in a 200 µm longitudinal portion of the retinal section (excluding the site of injection) of *Rho*-/- or C57/Bl6 mouse retinas. No difference of transduction was observed between *Rho*-/- and WT mice. In older animals (4.9 months, Figure 3d and d′), we obtained very few transduced photoreceptors in *Rho*-/- mice and some transduction in WT mice. To potentially reveal low GFP-expressing cells, we performed immunolabeling against GFP using a secondary antibody with a red fluorophore (Alexa633) to evidence cells not detected by GFP. We observed that red cells were also positive for GFP. In advanced degeneration processes (see Figure 3d), the LV-VSVG-Rhop-GFP vector conserved the same tropism as observed after injection into the normal retina, but with less efficiency.

**Figure 4:**
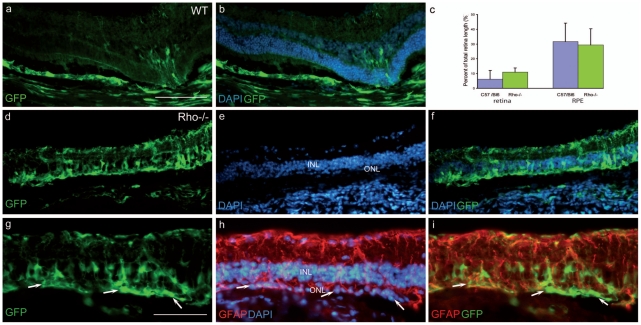
The LV-VSVG-EFs-GFP transduces cells of the inner nuclear layer and photoreceptors in the degenerating retina. In control animals (5.4 months), the vector transduces cells (green) in the neuroretina only at the injected area (a, b: GFP and DAPI staining). Note that RPE cells are well transduced (a, b). (c) The extension of the transduced area in the neuroretina is small (left columns) both in wild-type and *Rho*-/- mice (n = 5 for each group). The vector has a better efficacy on RPE cells (right columns). In 4.9 month-old Rho-/- mice, the vector diffuses into the retina and targets cells with different morphologies in the INL (d, f, g, i) as well as in the ONL (g, h, i, arrows). DAPI staining of (d) and (g) are presented in (e or h). (f) merged pictures of (d) and (e). (h) Immunolabelling for the Müller glial protein GFAP (red). (i) Very rare GFP-positive cells were positive for GFAP (red). INL: inner nuclear layer, ONL: outer nuclear layer. Calibration bar in (a): 100 µm (for a, b, d, e and f). Calibration bar in (g): 50 µm (for g, h and i).

Another vector bearing a ubiquitous promoter, a short fragment of the Elongation Factor1 promoter (LV-VSVG-EFs-GFP), was injected into the same model 1) to verify that this lack of photoreceptor transduction was not a problem of gene expression due to the health of the photoreceptors (decrease of rhodopsin promoter activity for instance), and 2) to determine whether other cell types can be reached by the vector when the OLM shows a disruption of the ZO-1 expression. The vector was tested in late-stage *Rho*-/- degenerating retinas and in age-matched WT controls. For all animals, the largest transduced area was observed in the RPE layer confirming previous data showing that the lentiviral vector carrying a ubiquitous promoter targets mainly RPE cells[5], [6]. The size of the transduced region of the RPE layer or the degenerating retinas of the *Rho*-/- mice was not different in comparison to normal transduced retinas (see graph in Figure 4c). This result shows that this vector has the same diffusion capacity in the wild-type or the diseased retina. Interestingly, in contrast with the control group, the *Rho*-/- retinas contained several GFP-positive cells in the neuroretina (but in the injection site near the needle tract only). We identified a significant number of GFP positive cells in the ONL in the transduced area of aged *Rho*-/- animals (Figure 4 g, h, and i, arrows), which was unexpected in view of the results we obtained with the LV-VSVG-Rhop-GFP. This suggests that the EFs promoter is more efficient than the *Rhodopsin* promoter to drive transgene expression in the photoreceptors, during advanced stage of the degeneration in the *Rho*-/- mice. This reduced activity of the *Rhodopsin* promoter may be the consequence of the poor photoreceptor health at this stage of degeneration in the *Rho*-/- mouse. We also checked whether cones were transduced by the vector, but only a very weak expression for GNAT2 or RXRγ was detected and rendered the co-localization detection problematic. Nonetheless, rare cells positive for GFP and GNAT2 were observed (data not shown) revealing that the EFs promoter is active in cones and that GFP positive cells in the ONL could also be cones.

Beside these photoreceptors, some of the GFP positive cells in the INL have a Müller cell appearance (Figure 4d and g). To confirm that some glia cells were targeted, we performed immunolabelling for GFAP and observed certain cells expressing both GFP and GFAP (Figure 4h and i). In addition, the high expression level of GFAP in the *Rho*-/- retina at this stage of the degenerating process attests of a gliosis process as previously described[22]. We also observed an increased GFAP immunolabeling at the OLM level (Figure 4h and i) revealing an important remodeling of this membrane during the degenerating process. A similar gliosis was detected in the retina of another mouse model of retinal degeneration, the *Rd1* mouse (Figure S1) suggesting that photoreceptor loss often produces a reactive gliosis.

Few cells in the INL with a bipolar morphology were positive for GFP, but not for the Go protein, normally expressed by wild-type bipolar cells (data not shown). We attempted to investigate other markers, such as PKCα. However, a strong decrease of the expression of this protein is observed in this model and renders co-localization with GFP difficult (data not shown). Other works have also described a reduction of PKCα during the retinal degeneration of other models[26], probably due to a lack of normal inputs from the photoreceptors.

**Figure 5:**
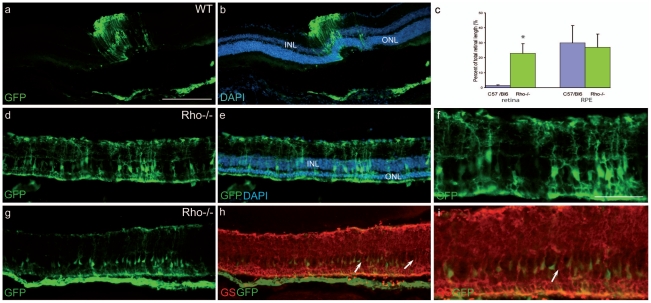
In the degenerating *Rho*-/- retina, the LV-Mok-CMV-GFP preferentially transduces Müller cells in an extended region of the retina. In control animals (WT, 3.3 months), the vector targets cells (green) only at the injected area (a, b with DAPI). (c) The LV- Mok-CMV-GFP vector transduces a large portion of the *Rho*-/- retina but not of the wild-type retina (compare blue columns). The extension of the transduction is similar in the retina and the RPE layer and represents around 25–30% of the retina. In the 2.4 month-old *Rho*-/- retinas, the vector infects many cells with a Müller morphology (d, e, f, g with DAPI). The RPE cells are not present in this picture. (f) shows a magnification of (d) to better show the Müller cell morphology of the transduced cells. (g) another example of transduced cells in the *Rho*-/- retina where both RPE cells and cells with a Müller cell morphology (arrow) are targeted (the brightness of RPE labelling partially hides the Müller cells). (h) many of the transduced cells express also glutamine synthetase (GS, red), specific of Müller cells. Few GS-positive cells do not express GFP (arrows). (i) The magnification of picture (h) reveals that the great majority of glutamine synthetase-positive cells were transduced. Note that few cells show no green signal in the cell body (arrow). INL: inner nuclear layer, ONL: outer nuclear layer, *: P = 0.014. Calibration bar in (a): 100 µm (for a, b, d, e, g and h). Calibration bar in (f): represents 50 µm for (f) and 54 µm for (i).

These results show that during the degenerating process the diffusion capacity of the lentiviral vector pseudotyped with the VSVG envelope is almost not changed. Nonetheless, we observed that some Müller cells can be targeted. Because a strong remodeling of glial cells occurs, we were interested to evaluate whether an efficient gene delivery could be achieved in these cells during retinal degeneration. To optimize the transduction of Müller cells, we changed the vector envelope by replacing VSVG by the Mokola virus envelope. Additionally, we changed the promoter for the CMV promoter, also shown to be active in Müller cells[27]. Indeed, previous results performed in the brain have shown that Mokola-pseudotyped lentiviral vectors can efficiently transduce glial cells[25], [28]. Adult control and mid-stage degeneration Rho-/- mice were injected with the LV-Mok-CMV-GFP vector. In the wild-type animals, the neuroretina is only transduced at the injection site and the expression is mainly restricted to a large percentage of the RPE cells and encompasses the size of the bleb performed during the injection (Figure 5a) as previously described[1], [29], thus very similar to the VSVG pseudotyped LV. A large portion of the RPE layer is targeted by the vector. By contrast in the *Rho*-/- retina, the behavior of the LV-Mok is very different in comparison to LV-VSVG. The Mokola vector transduces a large area of the neuroretina in the *Rho*-/- and only few cells in the WT animal, 23±6.5% (n = 5) of the retina surface versus 1.5±0.3% (n = 4) respectively (P = 0.014, Figure 5c). In the diseased retina, in addition to the transduction of RPE cells, almost all of the transduced cells have a Müller cell morphology (Figure 5d, f) and all of the targeted cells express glutamine synthetase, an enzyme only expressed in this cell type (Figure 5h and i). We estimated the percentage of Müller cells transduced by this vector and observed that 79±7.2% (n = 3) of the glial cells express GFP. The extension of the transduced retina area is comparable to the size of the RPE layer targeted by the virus revealing the ability of the LV-Mok to target the glial cells of the neuroretina in a diseased retina.

## Discussion

The present study shows that the lentiviral vector may have different capacity to transduce cells during the course of retinal degeneration. Indeed, retinal remodelling during the disease modifies the tropism of the LVs. Consequently, the use of different envelopes and promoters allows to play with different targets in the degenerating retina. Using specific Mokola-pseudotyped LV, Müller cells can be preferentially targeted during retinal degeneration along with RPE cells.

During retinal degeneration, the disruption of ZO-1 labelling integrity suggests that the OLM is altered rendering possible the entry of entities into the retina that are normally blocked in the healthy retina by this barrier. Indeed, ZO-1 is a major stabilization actor of the OLM connexion. In the *Rho*-/- mice, previous studies showed that the decreased expression of ZO-1 is also paralleled by a decrease of β-catenin association which is normally bound to ZO-1[20]. But the overall results were surprising in the late stage *Rho*-/- mice. Very few photoreceptors express GFP when using the LV with the *Rhodopsin* promoter suggesting that the vector diffuses less in the degenerating retina. However, the use of the ubiquitous promoter EFs shows that near the injection site some photoreceptors can be transduced suggesting that in the *Rho*-/- mice the *Rhodopsin* promoter activity may change during the course of retinal degeneration or that non-detectable cones can be transduced. Even if some photoreceptors can be targeted with the LV-EFs-GFP vector, the number of cells that can be transduced remains very low. This limited photoreceptor transduction is probably due to a change of OLM structure and glia paralleling ZO-1 expression change.

Indeed at a late stage of *Rho*-/- retina degeneration, an increased GFAP expression was observed with many processes surrounding the remaining photoreceptors (see Figure 4) suggesting a transformation of the OLM probably by reinforcing the barrier effect of the Müller cells endfeet. Previous studies have shown that during retinal degeneration, the Müller cells present a marked modification in protein expression[17], [21], beginning to express filament protein such as GFAP and also vimentin that may change the structure density of the cells. An interesting work has demonstrated that the integration of primary fœtal transplanted retinal cells is drastically reinforced when both vimentin and GFAP are missing in the Müller cells (GFAP-/-::Vimentin-/- mice), whereas a reduced migration is seen when either GFAP or vimentin alone is suppressed[30]. Our experiments suggest that at a later retinal degeneration stage, after the alteration of the ZO-1 structure, gliosis may impair VSVG-pseudotyped lentiviral vector diffusion.

In *Rho*-/- retinas, following the defect of ZO-1 expression, some other components of the extracellular matrix may compensate for this function loss. The ability of the vector to diffuse (or not) may thus be a question of timing during the course of OLM remodelling. CD44 or Neurocan are candidates of the extracellular matrix (ECM) that may enhance the barrier effect. These proteoaminoglycans were shown to impair the migration of transplanted retinal progenitor cells from the subretinal space towards the retina[23]. The Crumb protein was also shown to be an important protein for the OLM barrier effect on transplanted cell integration[22]. So far, the potential barrier influence of these components of the ECM on viral particle diffusion is unknown and merits to be investigated. Nonetheless, for certain approaches, the opening of this barrier seems to have to be limited in time to prevent retina cell alteration[31].

The lentiviral vector with the VSVG envelop was already shown to target Müller cells in the rat retina, when the vector contains the GFAP promoter[7]. Surprisingly, the presence of the CMV promoter does not allow expression of the reporter transgene in Müller cells[7]. In this cited study, the LV-GFAPp-eGFP vector pseudotyped by the VSVG envelope transduces a very large proportion of Müller cells (more than 90%) when subretinally injected at PN21 in normal and diseased retina (the S334Ter+/- rat which bears a dominant mutation in the *Rhodopsin* gene). The advantage of the LV-Mok vector described in our study is to target Müller cells only in a diseased retina, even using a ubiquitous promoter.

Other vectors were developed to target the Müller cells such as Adenovirus-derived vectors. The Ad5/F37 largely diffuses in the retina and transduces around 11% of the cells in the inner nuclear layer where Müller cells reside[32]. Using a directed evolution approach to select vectors that efficiently transduce primate astrocytes *in vitro*, Klimczak et al. identified that the AAV variant ShH10 has a great capacity to infect Müller cells *in vivo* when injected into the vitreous[33]. This elegant study reveals that more than 20% of the Müller cells can be transduced by such delivery way. This variant is close to the AA2/6 serotype, which also specifically transduces activated Müller glia cells (but with a lower efficacy than the cited above serotype), when the transgene expression is driven by the GFAP promoter[34]. The added-value of these vectors and approach is to avoid deleterious side effects with subretinal injections that are much more delicate in a degenerating retina. However, the delivery efficiency needs to be improved.

The use of the Mokola envelope redirects the LV targeting to a large proportion of Müller cells over an important distance corresponding to the bleb area (this probably through the endfeet which are in the vicinity of the RPE). This characteristic is seen only in the degenerating retina. This extended area of glial cell transduction might be interesting for using this vector for therapeutic applications. Indeed, targeting Müller cells may serve to deliver neurotrophic substances to support the survival of photoreceptor cells during retinal degeneration. In the context of such strategy so far, gene transfer approaches mainly aim to target the RPE cells. However, a constant fluid flux from the vitreous towards the choroid exists. In consequence, the trophic factor engineered in RPE cells is secreted against this flux which could limit its efficacy. In contrast, a trophic factor released by Müller cells may better reach the photoreceptors. Very interesting works recently demonstrated the usefulness of this approach in two different models of retinal degeneration using AAV vectors[35], [36]. Neurotrophic factor delivery may also be used in combination with gene replacement strategy. In addition, in severe forms of photoreceptor loss such as early forms of LCA and RP, such gene transfer can support the survival of the photoreceptors until the benefit of the transgene replacement occurs[37]. Other applications of our results could be the control of the Müller cell structure to allow a better integration of transplanted retinal cells derived from stem cells, for instance by reducing gliosis, which can be achieved by siRNA transfer[38].

Most of the studies on vector tropism were undertaken in normal retinas or at an early stage of retinal degeneration and little is known concerning the ability of the vectors to transduce retinal cells at advanced stages of retinal degeneration. We show that the Mokola vector can efficiently transduce Müller cells in a diseased retina while it had been described as a very specific tool to target RPE cells in a normal rat retina[1], [29]. Similarly, the *Rhodopsin* promoter surprisingly appeared less efficient than the EFs promoter to drive transgene expression in photoreceptors in a degeneration context, this will probably be true for any vector used, i.e. LV, AAV or AdV. In this regard, the overall meaning of our results underlines the importance of evaluating gene transfer tools in the final context of use since the environment as well as the target cells may be subjected to various modifications that will impact on the transduction capacities.

## Materials and Methods

### Vector production

The EFS-GFPII transgene cassette was obtained by subcloning hrGFPII (Agilent Technologies, Santa Clara, CA) in the Hlox-EFS-GFP backbone[5] after discarding the GFP gene. The Rho-GFP transgene plasmid was described in Kostic *et al.* [*5*]. Production of LV-VSV-G–Rhop or EFS-GFPII by cotransfection in 293T cells was performed as described in Bemelmans et al.[9]. The CMV-GFP cassette is described in[39]. Production of the Mokola-pseudotyped LV-CMV-GFP was also performed by triple transfection but using the Mokola plasmid[29] instead of the pMD.G encoding the VSV-G protein. Quantifications of the vector stocks were measured using the RETRO-TEK HIV-1 p24 Antigen ELISA kit (ZeptoMetrixCorporation, Buffalo, NY USA) according to the manufacturer's instructions.

### Ethic statement

The animals were handled in accordance with the statement of the "Animals in Research Committee" of the Association for Research in Vision and Ophthalmology, and protocols followed the national regulations (OFEV) and were approved by the local institutional committee, the “Service de la consommation et des affaires vétérinaires du canton de Vaud” (autorisation VD#1367.3).

### Animal procedure

Rhodopsin knockout mice were derived from Rpe65-/-::Rho-/- mice (Professor C. Grimm, University of Zürich) and crossed in the C57/Bl6 background. Each eye was subretinally injected with 150 ng of vector in 2 microliters as described previously[9]. For immunohistochemical procedures, the eyes were collected 1 week after the injection, cauterized to indicate the orientation, fixed in 4% paraformaldehyde, washed in PBS and then immerged in 30% sucrose overnight. The eye is then embedded in albumin from hen egg white (Fluka, Buchs, Switzerland) and cut into 14 µm cryostat sections.

### Immunohistochemistry

For immunohistochemistry sections were incubated with the first antibody overnight at 4°C in PBS containing 0.2% TritonX-100 and 10% normal horse serum. Sections were then washed in PBS and incubated with the secondary antibody at 37°C for 1 hour. The following primary antibodies were used: goat polyclonal anti-GFP for eyes injected with LV-Rho-GFP (1/3000, Abcam), mouse monoclonal anti-ZO-1 (ZO1-1A12, 1/60, Invitrogen, Camarillo, CA) and rabbit polyclonal anti-glutamine synthetase (1/5000, Sigma-Aldrich). For visualization, fluorescence-labelled secondary antibodies (Alexa-488 and Alexa-633; Molecular Probes) were used. Sections were counterstained with DAPI (Molecular Probes) and mounted under coverslips with Mowiol 4–88 Reagent (VWR International AG, Lucerne, Switzerland).

### Quantification and statistical analysis

To evaluate the potency of the vectors to spread in the subretinal space and in the retina, we measured near the injection site the maximum length of the area comprising transduced cells. The length of transduced RPE estimates the size of the injection bleb while the length of transduced retina cells reflects its efficiency to penetrate through the retinal layers. The values were expressed as percentage of total retinal length. If the size of both transduced RPE and transduced retina are similar, the tested vector shows the capacity to transduce retinal cells. If the size of GFP-positive retina is much smaller than the GFP-positive RPE region, the tested vector penetrated into the retina only because of the injury produced by the needle.

For statistical analyses, groups were compared by an unpaired Student T test using the Excel software (Microsoft, WA).

## Supporting Information

Figure S1 **Gliosis occurs during retinal degeneration of the** ***Rd1*** **mouse retina.** The mouse bears a mutation in the *Pde6b* gene leading to phototransduction inhibition and photoreceptor loss. At P12, GFAP (red) expression is mainly detected in the inner part of the retina. At p15, the gliosis increases when several layers of photoreceptors were already lost (compare the ONL size with WT retina). Magnification: 200x.
(PDF)
