# Supplementary figures and images for: Retinal Degeneration Progression Changes Lentiviral Vector Cell Targeting in the Retina
Source: PLoS One. 2011 Aug 25;6(8):e23782. doi: 10.1371/journal.pone.0023782 (PMC3161995; doi:10.1371/journal.pone.0023782)

**P10 *Rd1***

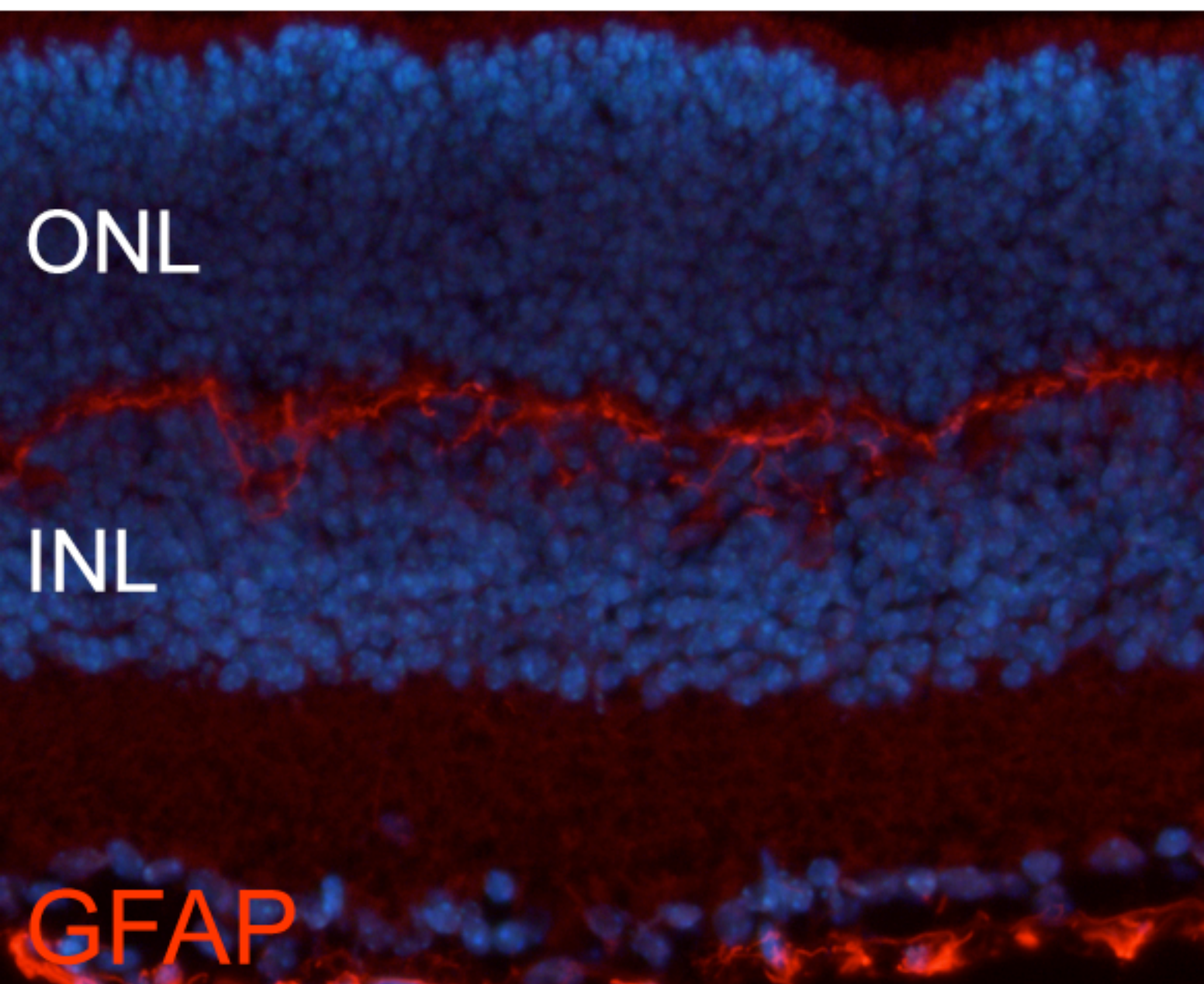

**P12 *Rd1***

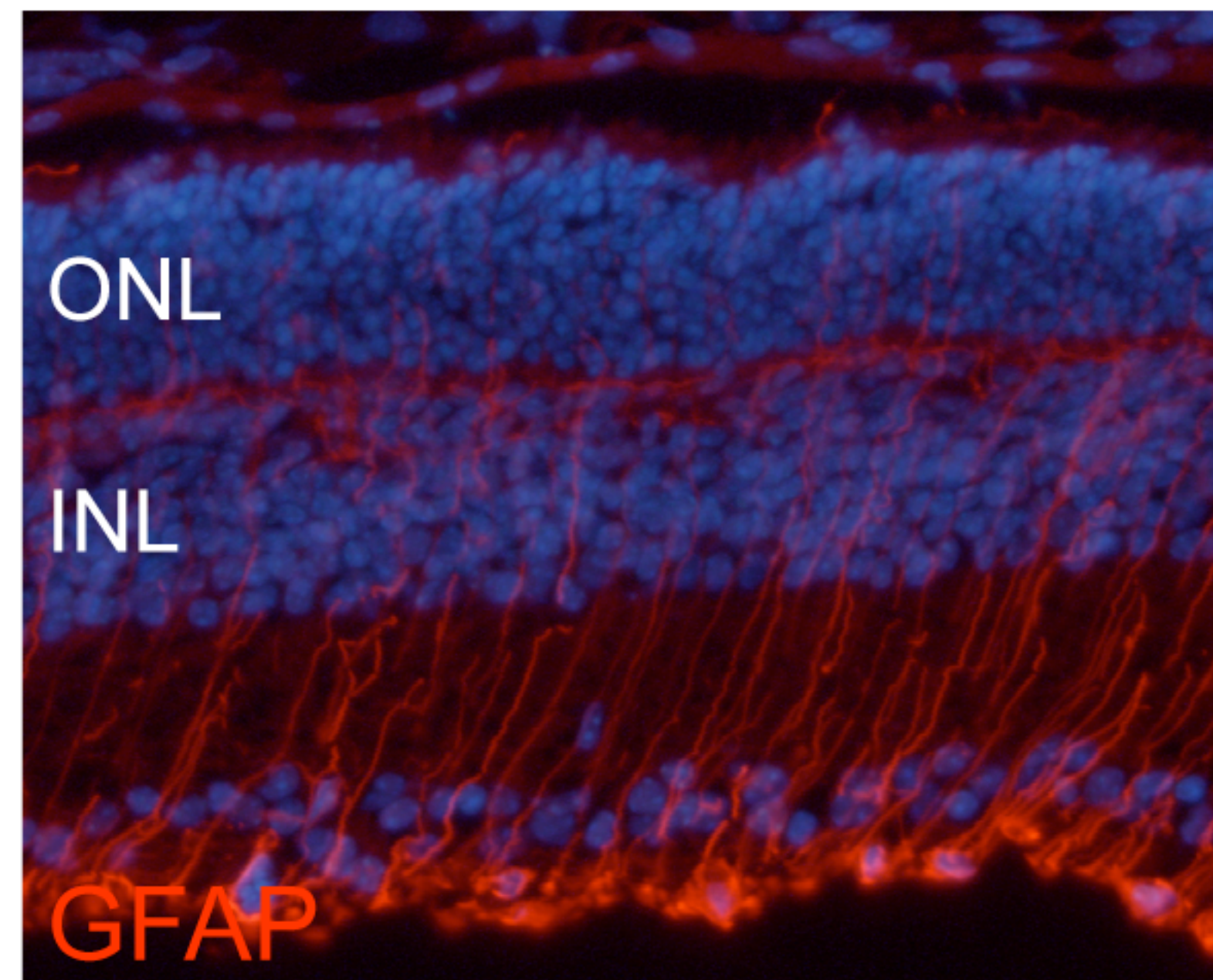

**P15 *Rd1***

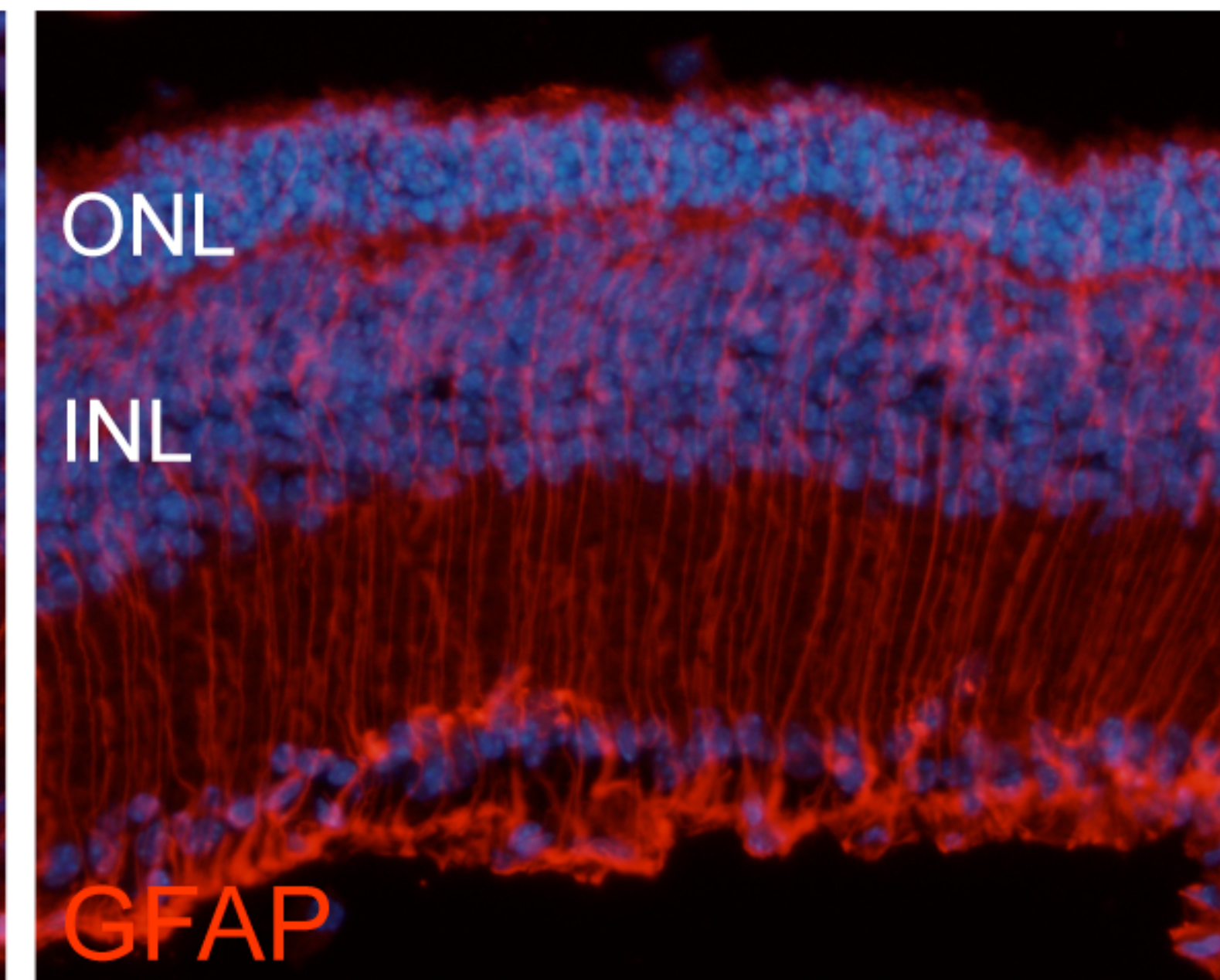

**P15 WT**

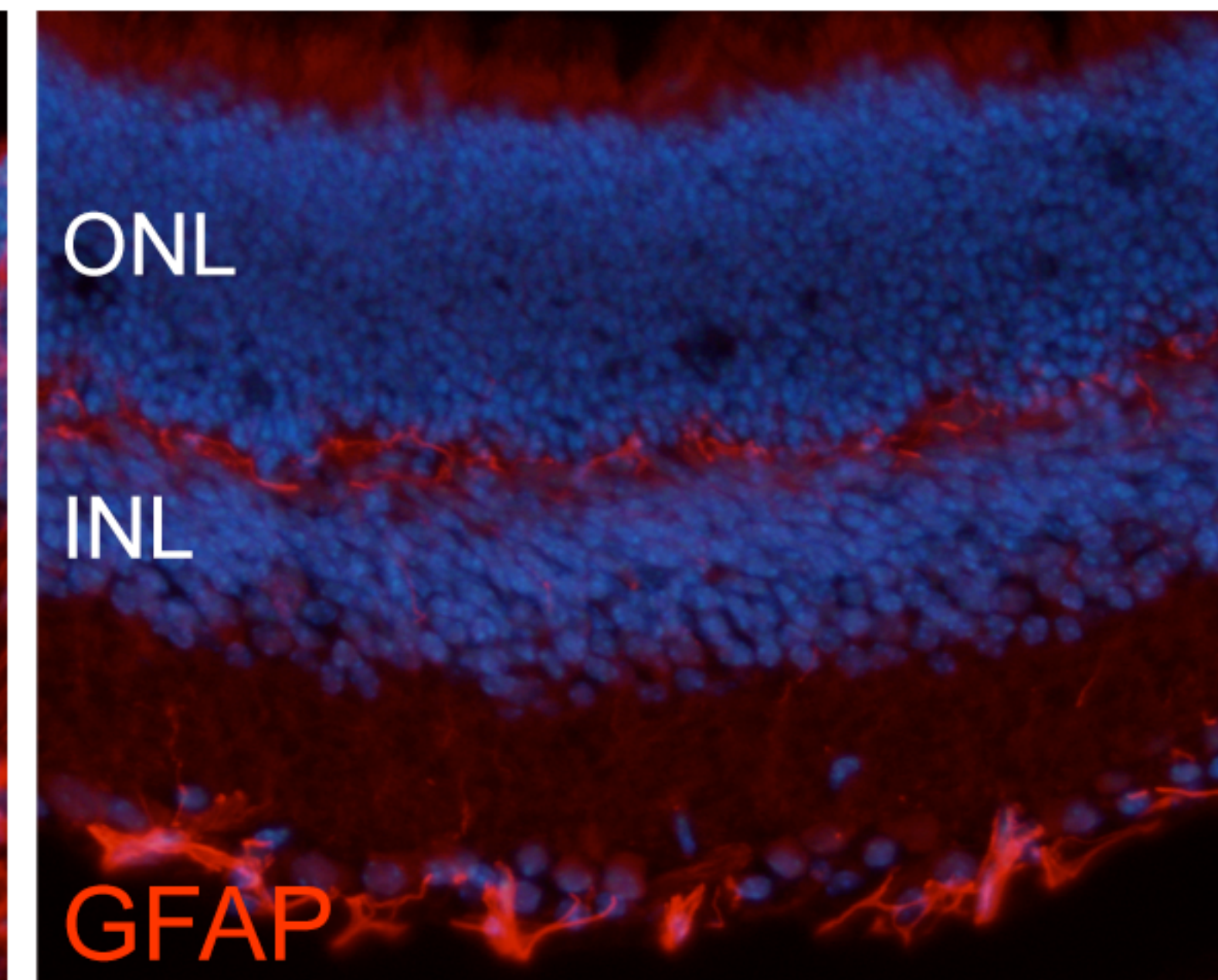

Supplement: Figure S1 — Gliosis occurs during retinal degeneration of the Rd1 mouse retina. The mouse bears a mutation in the Pde6b gene leading to phototransduction inhibition and photoreceptor loss. At P12, GFAP (red) expression is mainly detected in the inner part of the retina. At p15, the gliosis increases when several layers of photoreceptors were already lost (compare the ONL size with WT retina). Magnification: 200x. (PDF) [file pone.0023782.s001.pdf]
